# Surveillance for adverse events following immunization with DTaP-containing combination vaccines in Linping, China, 2019–2022

**DOI:** 10.3389/fpubh.2024.1278513

**Published:** 2024-03-26

**Authors:** Qinghua Chen, Chuandi Zhang, Chunmei Ye, Junwei Zhu, Jie Shen, Chang Zhu, Pai Yang, Tiane Liu, Yuyang Xu

**Affiliations:** ^1^Department of Expanded Program on Immunization, Linping District Center for Disease Control and Prevention, Hangzhou, Zhejiang, China; ^2^Department of Public Health, Linping District Hospital of Integrated Traditional Chinese and Western Medicine, Hangzhou, Zhejiang, China; ^3^Department of Expanded Program on Immunization, Hangzhou Center for Disease Control and Prevention, Hangzhou, Zhejiang, China

**Keywords:** DTaP, DTaP-Hib, DTaP-IPV/Hib, AEFI, reporting rate

## Abstract

**Background:**

The DTaP-Hib and DTaP-IPV/Hib combination vaccine can be used as a substitute for the diphtheria, tetanus, and acellular pertussis combined vaccine (DTaP). We aimed to evaluate the safety of multi-component vaccines containing DTaP by analyzing the reporting rates and characteristics of adverse events following immunization (AEFIs) in Linping District during the years 2019 to 2022.

**Methods:**

We obtained data of AEFI and vaccination from the National AEFI Surveillance System of China and Zhejiang Municipal Immunization Information Management System, respectively, during 2019–2022 for a descriptive, epidemiological analysis.

**Results:**

The total number of AEFI reported following vaccinations with DTaP-containing combination vaccines was 802 in Linping District from 2019 to 2022. The overall reporting rates of AEFIs following DTaP, DTaP-Hib, and DTaP-IPV/Hib vaccinations were 445.72 (537 cases), 536.29 (45 cases), and 306.13 (220 cases) per 100,000 doses in Linping District from 2019 to 2022, respectively. Only one case of a serious AEFI following DTaP vaccination, with a reporting rate of 0.83 per 100,000 doses. The composition ratio of vaccine product-related reactions for DTaP, DTaP-Hib, and DTaP-IPV/Hib were 99.81, 97.78, and 100.00%, respectively. The composition ratio of coincidental events for DTaP and DTaP-Hib were 0.19 and 2.22%, respectively. The reporting rates of total AEFIs for DTaP-IPV/Hib were lower than for DTaP. The reporting rate of local induration for DTaP-Hib was lower than for DTaP, and the reporting rates of local redness & swelling and local induration for DTaP-IPV/Hib were both lower than for DTaP. DTaP-IPV/Hib had a higher proportion of AEFIs in first quarter compared to DTaP. The reporting rate after the second dose of DTaP-Hib was higher than that of DTaP, and the reporting rates of AEFIs after the first dose and third dose of DTaP-IPV/Hib were lower than DTaP.

**Conclusion:**

The reported AEFIs to multi-component vaccines containing DTaP components during 2019–2022 in Linping District were mainly mild vaccine reactions. DTaP-containing combination vaccines demonstrated a good safety profile.

## 1 Introduction

Pertussis (whooping cough), Diphtheria, and Tetanus, as vaccine-preventable diseases (VPDs), are seriously harmful to children’s health. Pertussis, known as “whooping cough (cough for 100 days)” in folk medicine, is an acute respiratory infectious disease. Prior to widespread coverage of the pertussis vaccine (PV), pertussis was the primary cause of death among infants and young children (1). A study over the lifetime of 40 birth cohorts from 1978 to 2017 in China show that pertussis cases and deaths were decreased by an estimated 92.57 and 97.43% with the widespread use of PV (2). Diphtheria, one of the VPDs, mainly includes respiratory diphtheria and cutaneous diphtheria, affecting respiratory mucosa and skin. The global case fatality rate is around 10%, and it keeps in severe cases (3). With diphtheria vaccination, the incidence of disease has decreased dramatically (4). Tetanus is a disease that mainly affects the central and peripheral nervous systems. World Health Organization (WHO) estimates that approximately 34,000 neonates died of neonatal tetanus in 2015, which represents a 96% reduction since 1988 (5). Previous research shows that the tetanus vaccination have resulted in the elimination of neonatal tetanus in the developing countries (6). Preventive vaccination is the most direct, effective, and cost-efficient measure for controlling infectious diseases. At present, there are three kinds of inactivated vaccines used to prevent pertussis, diphtheria, and tetanus in Linping District, which are the diphtheria, tetanus, and acellular pertussis combined vaccine (DTaP), the diphtheria, tetanus, and acellular pertussis-haemophilus influenza type b combined with vaccine (DTaP-Hib) and the diphtheria, tetanus, and acellular pertussis-inactivated poliovirus-haemophilus influenza type b combined vaccine (DTaP-IPV/Hib).

The “Vaccine administration law of the People’s Republic of China” classifies vaccines used domestically into two main categories: the National Immunization Program (NIP) vaccines and non-NIP vaccines. NIP refers to a schedule of government-determined vaccines that is administered to the population at specific times (7). Over the past 3 years, the NIP vaccination rate in Linping has remained above 97%. The high vaccination rate of NIP vaccines is achieved through significant financial support from the government, effectively controlling the prevalence of VPDs. Among these, the DTaP is one of the most widely used and extensively administered vaccines in the Linping District. According to the record, DTaP was introduced in 1999 in Hangzhou, completely replaced the diphtheria, tetanus, and whole-cell pertussis combined vaccine (DTwP) in 2011 (8). The main components of DTaP were diphtheria toxoid and tetanus toxoid combined with acellular pertussis. The DTaP vaccination schedule of infants and young children in Linping District aligns with that of Chinese mainland. In Linping District, the vaccination rates achieved with four doses of the DTaP vaccination in childhood have been more than 98% in recent years. Non-NIP vaccines serve as effective supplements to NIP vaccines and have gained increasing attention as an effective means of disease prevention and control. As public health awareness has increased, the size of vaccinated populations has grown substantially, leading to a significant rise in the use of non-NIP vaccines. These include the DTaP-Hib and the DTaP-IPV/Hib in the Linping District, which have replaced DTaP in China or other countries (9–12), used for the prevention of VPDs such as Pertussis, Diphtheria, Tetanus, Poliomyelitis, and *Haemophilus influenzae* type b. Studies have shown that multi-component vaccines have seen significant increases in usage nationwide, avoiding the pain caused by multiple injections of component vaccines, reducing physical and human resources costs, and lowering expenses. This approach also reduces visits to vaccination clinics to avoid the cross-contamination with other intranasal spray vaccines, leading to improved compliance among recipients and increased immunization coverage (13, 14).

Vaccines are usually administered to healthy people, including entire birth cohorts of infants and in vast numbers. However, adverse events following immunization (AEFI) may occur in the recipient following the use of any vaccine, the most severe cases are extremely rare and are usually mild and self-limiting (15–17). Scientific studies demonstrated that strong antibody-based and cellular immunoprotective stimulation against each component of pertussis typically requires a combination of several adjuvants (18, 19). Due to the presence of aluminum hydroxide adjuvant, there is an increased reporting rate of injection site reactions in recipients with vaccination (20, 21). A major challenge to the safety of the DTaP combination vaccine is that the vaccine needs to be administered in early infancy. To assess the safety and effective administration of vaccines, especially when new vaccines are introduced in any country, WHO recommends that countries should establish effective AEFI reporting programs (17). The AEFI surveillance are conducted by either applying a passive or active approach to monitor vaccine safety in various countries (22–24). However, systematic research is lacking on comparison of AEFI at the multi-component vaccines containing DTaP component. Consequently, there is a great emphasis on the safety of administering these combination vaccines. This study aims to compare and analyze the characteristics of AEFIs reported from DTaP, DTaP-Hib, and DTaP-IPV/Hib vaccinations in the Linping District during the years 2019 to 2022. The goal is to evaluate the safety of vaccines containing DTaP component.

## 2 Data and methods

### 2.1 Data source

The adverse event case data following the administration of DTaP, DTaP-Hib, and DTaP-IPV/Hib vaccines in Linping District from January 1, 2019, to December 31, 2022, were collected through the China AEFI Surveillance and Management Information System. The number of doses administered for DTaP, DTaP-Hib, and DTaP-IPV/Hib vaccinations in the preventive immunization clinics of Linping District during the same period was collected through the Zhejiang Province Comprehensive Management Information System for Vaccines and Immunization (data downloaded on June 5, 2023). The annual doses of the DTaP vaccine in 2019, 2020, 2021, and 2022 were 41,728, 31,458, 26,861, and 20,432, respectively, for a total of 120,479 doses. The annual doses of the DTaP-Hib vaccine were 1,920, 2,375, 2,113, and 1,983, respectively, for a total of 8,391 doses. The annual doses of the DTaP-IPV/Hib vaccine were 14,216, 17,434, 19,718, and 20,496, respectively, for a total of 71,864 doses.

### 2.2 Vaccine types and immunization schedule

Diphtheria, tetanus, and acellular pertussis combined vaccine is a national NIP vaccine, the vaccination schedule for infants and young children in Linping District aligns with that of Chinese mainland, consisting of four 0.5 mL doses of DTaP administered at 3, 4, 5, and 18 months of age (intramuscular injection). DTaP-Hib and DTaP-IPV/Hib are non-NIP vaccines voluntarily and optionally administered at an individual’s expense. They are combination vaccines containing acellular pertussis component and are used as substitutes for DTaP. The immunization procedure for DTaP-Hib is consistent with that of domestic DTaP, consisting of four 1.0 mL doses (intramuscular injection), while the DTaP-IPV/Hib vaccine is recommended for administration as three 0.5 mL doses at 2, 3, and 4 months of age to complete the primary course, followed by a booster at 18 months (intramuscular injection).

### 2.3 AEFIs reporting and classification

According to national AEFI guidance, any AEFI should be reported mandatorily when it was detected by these authorized reporters, including health care facilities, Center for Disease Control and Prevention (CDC) at any administrative levels, adverse drug reaction monitoring agencies, and vaccine manufacturers executive staff. Additionally, the public or the caregivers could notify any of the above authorized reporters to report an AEFI. AEFI reports were gathered by local, county-level CDCs, which were responsible for completing AEFI case reporting cards and submitting data to the national AEFI surveillance system (25). A single AEFI report might be assigned more than one term and be referred to more than one suspected vaccine. In cases of co-administration of two or more vaccines at the same time in an individual, we attributed the reported AEFI to the reporter suspected vaccine according to the following principle: (1) The injection site reaction could be determined by the record of vaccination; (2) The systematic reactions could not be determined which vaccine was to be suspected when the co-administration occurred. In that case, we attributed the reported AEFI to all vaccines co-administrated.

An AEFI is defined as a reaction or an event following vaccination that is suspected to be related to the vaccination, according to the requirements of the national AEFI guidance, and supported by the Vaccine Administration Law of the People’s Republic of China. AEFIs are classified into vaccine product-related reactions (non-serious reaction and serious reaction), vaccine quality defect-related reactions, immunization error-related reactions, immunization anxiety-related reactions, and coincidental events (26). These are verified by AEFI monitoring professionals or diagnosed by a panel of experts investigating AEFIs following immunization. A non-serious reaction refers to a situation where no intervention is necessary or there is a physician visit or an event that interferes with daily activities or results in loss of working hours, including fever, local redness & swelling, and local induration. A serious reaction refers to with any untoward medical occurrence that results in death, hospitalization, prolongation of hospitalization, persistent or significant disability/incapacity, and life threatening conditions or birth defects.

### 2.4 Statistical analysis

The adverse event case data and the number of vaccine doses administered were processed using Microsoft Excel 2007. The annual reporting rate and composition ratio of AEFIs were calculated as follows: AEFIs Reporting Rate (/100,000 doses) = Number of AEFI Cases/Number of Vaccine Doses Administered × 100,000; AEFIs Composition Ratio (/100) = Number of AEFI Cases components/Number of whole AEFI Cases × 100. Data analysis was performed using SPSS 22.0, employing the *χ^2^* test for trend analysis and inter-group comparison, with a significance level of *α* = 0.05. The Bonferroni method was used for multiple (*k*) group comparisons, with a significance level of *α*’ = 0.05/*k*.

### 2.5 Ethics approval

No ethics approval was required for this study as it is a systematic review using preexisting, publicly published data.

## 3 Results

### 3.1 AEFIs reporting and classification (clinical diagnosis)

The total number of AEFI reported following vaccinations with DTaP-containing combination vaccines in Linping District from 2019 to 2022 was 802. The overall reporting rates of AEFIs following DTaP, DTaP-Hib, and DTaP-IPV/Hib vaccinations in Linping District during 2019–2022 were 445.72 per 100,000 doses (537 cases, 120,479 doses), 536.29 per 100,000 doses (45 cases, 8,391 doses), and 306.13 per 100,000 doses (220 cases, 71,864 doses), respectively. The annual reporting rate of AEFIs for DTaP increased from 337.90 per 100,000 doses to 636.26 per 100,000 doses, showing a statistically significant upward trend from 2019 to 2022 (*χ^2^* = 17.87, *p* < 0.001). There was no statistically significant difference in the reporting rate of AEFIs between DTaP and DTaP-Hib (*χ^2^* = 1.43, *p* = 0.232), but the reporting rate of AEFIs was higher for DTaP compared to DTaP-IPV/Hib (*χ^2^* = 22.37, *p* < 0.001). Most of the AEFIs reported following vaccines containing the DTaP component were non-serious. Only one case of a serious AEFI following DTaP vaccination, diagnosed as epilepsy, has been reported, with a reporting rate of 0.83 per 100,000 doses.

Regarding the types of AEFI reports, for DTaP and DTaP-Hib, the reporting rates of vaccine product-related reactions were 444.89 per 100,000 doses (536 cases, 99.81%) and 524.37 per 100,000 doses (44 cases, 97.78%), respectively. The one reported coincidental event for DTaP and DTaP-Hib was diagnosed as epilepsy and bronchopneumonia, respectively. For DTaP-IPV/Hib, all reported AEFI cases were vaccine product-related reactions. The reporting rates of vaccine product-related reactions for DTaP showed an upward trend from 2019 to 2022 (*χ^2^* = 17.31, *p* < 0.05). The reporting rates of vaccine product-related reactions for DTaP-IPV/Hib was lower than those for DTaP (*χ^2^* = 22.14, *p* < 0.001).

For the specific vaccine product-related reactions reported for DTaP, the reporting rates were as following: fever, 7.47 per 100,000 doses (nine cases, 1.68%); local redness & swelling, 305.45 per 100,000 doses (368 cases, 68.66%); local induration, 262.29 per 100,000 doses (316 cases, 58.96%); and rash, 1.66 per 100,000 doses (two cases, 0.37%). For DTaP-Hib, the reporting rates of local redness & swelling and local induration were 405.20 per 100,000 doses (34 cases, 77.27%) and 143.01 per 100,000 doses (12 cases, 27.27%), respectively. For DTaP-IPV/Hib, the reporting rates of fever, local redness & swelling, and local induration were 8.35 per 100,000 doses (six cases, 2.73%), 205.94 per 100,000 doses (148 cases, 67.27%), and 136.37 per 100,000 doses (98 cases, 44.55%), respectively. The reporting rates of local redness & swelling and local induration for DTaP and local induration for DTaP-IPV/Hib showed an upward trend (*p* < 0.05), while the reporting rate of fever for DTaP-IPV/Hib showed a downward trend from 2019 to 2022 (*χ^2^* = 6.61, *p* = 0.010). The reporting rate of local induration for DTaP-Hib was lower than DTaP (*χ^2^* = 4.35, *p* = 0.037), and the reporting rates of local redness & swelling and local induration for DTaP-IPV/Hib were both lower than DTaP (*χ^2^* = 16.66, 32.87, *p* < 0.001) (Table 1).

**Table 1:** Reporting rates (per 10^5^ doses) of DTaP, DTaP-Hib, and DTaP-IPV/Hib AEFIs by classification (clinical diagnosis) in Linping District, 2019–2022.

Classification (Clinical diagnosis)	*N* (Reporting rate)					*χ*^2^	*p*-value
	2019 Year	2020 Year	2021 Year	2022 Year	Total		
DTaP							
Vaccine product-related reaction	141 (337.90)	162 (514.97)	104 (387.18)	129 (631.36)	536 (444.89)	17.31	<0.001
Fever	4 (9.59)	5 (15.89)	0 (0.00)	0 (0.00)	9 (7.47)	3.27	0.071
Redness and swelling	113 (270.80)	100 (317.88)	75 (279.22)	80 (391.54)	368 (305.45)	4.12	0.042
Induration	67 (160.56)	102 (324.24)	62 (230.82)	85 (416.01)	316 (262.29)	24.76	<0.001
Rash	1 (2.4)	1 (3.18)	0 (0.00)	0 (0.00)	2 (1.66)	0.85	0.356
Coincidental event (Epilepsy)	0 (0.00)	0 (0.00)	0 (0.00)	1 (4.89)	1 (0.83)	—	—
DTaP-Hib							
Vaccine product-related reaction	3 (156.25)	19 (800.00)	13 (615.24)	9 (453.86)	44 (524.37)	0.74	0.389
Redness and swelling	2 (104.17)	15 (631.58)	10 (473.26)	7 (353.00)	34 (405.20)	0.66	0.416
Induration	1 (52.08)	4 (168.42)	4 (189.30)	3 (151.29)	12 (143.01)	0.66	0.417
Coincidental event (Bronchopneumonia)	0 (0.00)	0 (0.00)	1 (47.33)	0 (0.00)	1 (11.92)	—	—
DTaP-IPV/Hib							
Vaccine product-related reaction	19 (133.65)	68 (390.04)	73 (370.22)	60 (292.74)	220 (306.13)	3.83	0.050
Fever	3 (21.10)	3 (17.21)	0 (0.00)	0 (0.00)	6 (8.35)	6.61	0.010
Redness and swelling	13 (91.45)	44 (252.38)	48 (243.43)	43 (209.80)	148 (205.94)	3.61	0.057
Induration	5 (35.17)	28 (160.61)	34 (172.43)	31 (151.25)	98 (136.37)	6.51	0.011

### 3.2 Distribution of AEFIs by gender, age, and quarter

For DTaP AEFIs, the proportion of male and female cases was 55.87 and 44.13%, respectively, with a male-to-female ratio of 1.27: 1. The proportion of AEFIs in males was higher than in females (*χ^2^* = 14.78, *p* < 0.001). For DTaP-Hib, the proportion of male and female cases was 51.11 and 48.89%, respectively, with a male-to-female ratio of 1.05: 1. For DTaP-IPV/Hib, the proportion of male and female cases was 48.64 and 51.36%, respectively, with a male-to-female ratio of 0.95: 1. There was no statistically significant difference in the gender ratio between DTaP and the two alternative vaccines (*p* > 0.05).

Regarding age distribution, for DTaP AEFIs, the proportion of cases in the age groups 2–5, 6–17, and ≥ 18 months was 24.77, 23.28, and 51.96%, respectively. For DTaP-Hib, the proportion in the respective age groups was 28.89, 22.22, and 48.89%. For DTaP-IPV/Hib, the proportion in the respective age groups was 35.00, 15.45, and 49.55%. There were statistically significant differences in the distribution of AEFIs among the three vaccines across different age groups (*χ^2^* = 125.97, 7.80, 57.94, *p* < 0.05). According to the Bonferroni method, the significance level was adjusted to 0.0167 for pairwise comparisons among the three groups. DTaP and DTaP-IPV/Hib showed the highest proportion of AEFIs in the ≥18 months age group (*p* < 0.017). DTaP-Hib had a higher proportion of AEFIs in the ≥18 months age group compared to the 6–17 months age group (*p* < 0.017). DTaP-IPV/Hib showed the lowest proportion of AEFIs in the 6–17 months age group (*p* < 0.017). There was no statistically significant difference in the distribution of AEFIs among different age groups between DTaP and DTaP-Hib (*p* > 0.05), but DTaP-IPV/Hib had a higher proportion in the 2–5 months age groups (*χ^2^* = 8.15, *p* = 0.004) and a lower proportion of AEFIs in the 6–17 months age groups compared to DTaP (*χ^2^* = 5.76, 7.60, *p* < 0.05) (Table 2).

**Table 2:** Proportion of reported DTaP, DTaP-Hib, and DTaP-IPV/Hib AEFIs by gender and age in Linping District, 2019–2022.

Variable	*N* (%)		*χ*^2^	*p -value*	*N* (%)	*χ*^2^	*p*-value
	DTaP	DTaP-Hib			DTaP-IPV/Hib		
Gender							
Male	300 (55.87)	23 (51.11)	0.38	0.538	107 (48.64)	3.28	0.070
Female	237 (44.13)	22 (48.89)			113 (51.36)		
Months of age							
2	133 (24.77)	13 (28.89)	0.38	0.540	77 (35.00)	8.15	0.004
6	125 (23.28)	10 (22.22)	0.03	0.872	34 (15.45)	5.76	0.016
18	279 (51.96)	22 (48.89)	0.16	0.693	109 (49.55)	0.36	0.547

Regarding quarterly distribution, for DTaP, the reporting rates of AEFIs in the first, second, third, and fourth quarters were 169.06 per 100,000 doses, 522.57 per 100,000 doses, 592.27 per 100,000 doses, and 430.83 per 100,000 doses, respectively. For DTaP-Hib, the reporting rate in the respective quarters was 285.51 per 100,000 doses, 567.72 per 100,000 doses, 846.77 per 100,000 doses, and 293.54 per 100,000 doses. For DTaP-IPV/Hib, the reporting rate in the respective quarters was 224.63 per 100,000 doses, 371.57 per 100,000 doses, 373.96 per 100,000 doses, and 234.38 per 100,000 doses. There were statistically significant differences in the distribution of AEFIs for DTaP vaccine across different quarters (*χ^2^* = 64.24, *p* < 0.001). According to the Bonferroni method, the significance level was adjusted to 0.0125 for pairwise comparisons among the four groups. The reporting rate of AEFIs for DTaP was lowest in the first quarter (*p* < 0.013). There was no statistically significant difference in the distribution of AEFIs among different quarters between DTaP and DTaP-Hib (*p* > 0.05), but DTaP-IPV/Hib had lower reporting rates of AEFIs in the second, third, and fourth quarters compared to DTaP (*χ^2^* = 6.14, 11.16, 11.85, *p* < 0.05) (Table 3).

**Table 3:** Reporting rates (per 105 doses) of DTaP, DTaP-Hib, and DTaP-IPV/Hib AEFIs by quarter in Linping District, 2019–2022.

Variable	*N* (Reporting rate)		*χ*^2^	*p*-value	*N* (Reporting rate)	*χ*^2^	*p*-value
	DTaP	DTaP-Hib			DTaP-IPV/Hib		
1	43 (169.06)	4 (285.51)	1.03	0.518	35 (224.63)	1.57	0.210
2	188 (522.57)	14 (567.72)	0.09	0.764	72 (371.57)	6.14	0.013
3	189 (592.27)	21 (846.77)	2.46	0.117	71 (373.96)	11.16	0.001
4	117 (430.83)	6 (293.54)	0.85	0.355	42 (234.38)	11.85	0.001

### 3.3 Distribution of vaccine product-related reactions based on clinical diagnosis

Among vaccine product-related reactions, the reporting rates of fever between 37.6 and 38.5°C and fever above 38.6°C following DTaP vaccination were 6.64 and 0.83 per 100,000 doses, respectively. The reporting rate of fever between 37.6 and 38.5°C was higher than fever above 38.6°C (*χ^2^* = 5.44, *p* = 0.021). Following DTaP-IPV/Hib vaccination, the reporting rate of fever between 37.6 and 38.5°C was 8.35 per 100,000 doses, which was higher than DTaP (*χ^2^* = 6.99, *p* = 0.008).

Regarding the size of local redness & swelling, the reporting rates of local redness & swelling with diameters of ≤2.5, 2.6–5.0, and > 5.0 cm following DTaP vaccination were 9.13, 261.46, and 34.86 per 100,000 doses, respectively. Following DTaP-Hib vaccination, the respective reporting rates were 23.84, 274.10, and 107.26 per 100,000 doses. Following DTaP-IPV/Hib vaccination, the respective reporting rates were 13.92, 168.37, and 23.66. There were statistically significant differences in the reporting rates of local redness & swelling among the three vaccines based on different diameters (*χ^2^* = 456.73, 20.20, 156.77, *p* < 0.001). The highest reporting rate of local redness & swelling for DTaP was in the 2.6–5.0 cm diameter group, and the lowest was in the ≤2.5 cm diameter group (*p* < 0.001). Both DTaP-Hib and DTaP-IPV/Hib showed higher reporting rates of local redness & swelling in the 2.6–5.0 cm diameter group compared to the ≤2.5 and > 5.0 cm diameter groups (*p* < 0.017). The reporting rate of local redness & swelling with a diameter of >5.0 cm was higher for DTaP-Hib compared to DTaP (*χ^2^* = 10.39, *p* = 0.005), and the reporting rate of local redness & swelling with a diameter of 2.6–5.0 cm was lower for DTaP-IPV/Hib compared to DTaP (*χ^2^* = 17.24, *p* < 0.001).

Regarding the size of local induration, the reporting rates of local induration with diameters of ≤2.5, 2.6–5.0, and > 5.0 cm following DTaP vaccination were 66.40, 195.05, and 0.83 per 100,000 doses, respectively. Following DTaP-Hib vaccination, the respective reporting rates of local induration with diameters of ≤2.5 and 2.6–5.0 cm were 83.42 and 59.59 per 100,000 doses. Following DTaP-IPV/Hib vaccination, the respective reporting rates of local induration with diameters of ≤2.5, 2.6–5.0, and > 5.0 cm were 26.44, 108.54, and 1.39 per 100,000 doses. There were statistically significant differences in the reporting rates of local induration between DTaP and DTaP-IPV/Hib based on different diameters (*χ^2^* = 269.29, 99.37, *p* < 0.001). Both vaccines showed the highest reporting rates of local induration in the 2.6–5.0 cm diameter group and the lowest in the >5.0 cm diameter group (*p* < 0.001). The reporting rate of local induration with a diameter of ≤2.5 cm was higher for DTaP-IPV/Hib compared to DTaP (*χ^2^* = 13.97, *p* < 0.001). The reporting rates of local induration with diameters of 2.6–5.0 cm were both lower for DTaP-Hib and DTaP-IPV/Hib compared to DTaP (*χ^2^* = 7.74, 20.74, *p* < 0.05) (Table 4).

**Table 4:** Reporting rates (per 10^5^ doses) of DTaP, DTaP-Hib, and DTaP-IPV/Hib vaccine product-related reactions by clinical diagnosis in Linping District, 2019–2022.

Clinical diagnosis	*N* (Reporting rate)		*χ*^2^	*p*-value	*N* (Reporting rate)	*χ*^2^	*p*-value
	DTaP	DTaP-Hib			DTaP-IPV/Hib		
DTaP							
Fever (°C)							
37.6–38.5	8 (6.64)	0 (0.00)	—	—	6 (8.35)	6.99	0.008
≥38.6	1 (0.83)	0 (0.00)	—	—	0 (0.00)	—	—
Redness and swelling (cm)							
≤2.5	11 (9.13)	2 (23.84)	1.68	0.206	10 (13.92)	0.94	0.331
2.6–5.0	315 (261.46)	23 (274.10)	0.05	0.827	121 (168.37)	17.24	<0.001
>5.0	42 (34.86)	9 (107.26)	10.39	0.005	17 (23.66)	1.84	0.175
Induration (cm)							
≤2.5	80 (66.4)	7 (83.42)	0.34	0.562	19 (26.44)	13.97	<0.001
2.6–5.0	235 (195.05)	5 (59.59)	7.74	0.005	78 (108.54)	20.74	<0.001
>5.0	1 (0.83)	0 (0.00)	—	—	1 (1.39)	0.14	1.000

### 3.4 Distribution of AEFIs by occurrence time

Following DTaP vaccination, the proportion of reported AEFIs within ≤1 day, 2–5 days, 6–15 days, and > 15 days was 55.68, 14.71, 19.18, and 10.43%, respectively. Following DTaP-Hib vaccination, the respective proportions were 68.89, 8.89, 13.33, and 8.89%. The proportion of AEFIs reported within 2–5 days showed a decreasing trend from 2019 to 2022 (*χ^2^* = 4.50, *p* = 0.037). Following DTaP-IPV/Hib vaccination, the proportions were 58.64, 11.36, 20.45, and 9.55%, respectively. There were statistically significant differences in the distribution of AEFIs among the three vaccines based on different occurrence times (*χ^2^* = 370.40, 61.96, 185.02, *p* < 0.001). The proportions of AEFIs reported within ≤1 day for all three vaccines were higher than those reported within 2–5, 6–15, and > 15 days (*p* < 0.001). The proportion of AEFIs reported within 6–15 days following DTaP and DTaP-IPV/Hib were higher than those reported after >15 days (*p* < 0.013). There was no statistically significant difference in the distribution of AEFIs based on occurrence time between DTaP and DTaP-Hib, as well as DTaP-IPV/Hib (*p* > 0.05) (Table 5).

**Table 5:** Proportion of reported DTaP, DTaP-Hib, and DTaP-IPV/Hib AEFIs by onset time in Linping District, 2019–2022.

Onset time	*N* (%)					*χ*^2^	*p*-value
	2019 Year	2020 Year	2021 Year	2022 Year	Total		
DTaP							
≤1 day	92 (65.25)	81 (50.00)	51 (49.04)	75 (57.69)	299 (55.68)	1.38	0.239
2–5 days	27 (19.15)	23 (14.20)	14 (13.46)	15 (11.54)	79 (14.71)	2.96	0.086
6–15 days	20 (14.18)	28 (17.28)	29 (27.88)	26 (20.00)	103 (19.18)	3.18	0.074
>15 days	2 (1.42)	30 (18.52)	10 (9.62)	14 (10.77)	56 (10.43)	2.58	0.108
DTaP-Hib							
≤1 day	1 (33.33)	13 (68.42)	10 (71.43)	7 (77.78)	31 (68.89)	1.21	0.283
2–5 days	1 (33.33)	3 (15.79)	0 (0.00)	0 (0.00)	4 (8.89)	4.50	0.037
6–15 days	0 (0.00)	1 (5.26)	4 (28.57)	1 (11.11)	6 (13.33)	1.12	0.330
>15 days	1 (33.33)	2 (10.53)	0 (0.00)	1 (11.11)	4 (8.89)	0.88	0.397
DTaP-IPV/Hib							
≤1 day	13 (68.42)	39 (57.35)	33 (45.21)	44 (73.33)	129 (58.64)	0.75	0.385
2–5 days	2 (10.53)	10 (14.71)	9 (12.33)	4 (6.67)	25 (11.36)	1.16	0.311
6–15 days	4 (21.05)	7 (10.29)	23 (31.51)	11 (18.33)	45 (20.45)	0.92	0.376
>15 days	0 (0.00)	12 (17.65)	8 (10.96)	1 (1.67)	21 (9.55)	2.59	0.114

### 3.5 Distribution of AEFIs by number of vaccination doses

The reporting rates of AEFIs following one dose, two doses, three doses, and four doses of DTaP vaccination were 225.57 per 100,000 doses (10.99%), 295.52 per 100,000 doses (15.46%), 393.51 per 100,000 doses (21.60%), and 759.00 per 100,000 doses (51.96%), respectively. The reporting rates of AEFIs following one dose, two dose, and three doses of DTaP showed an increasing trend from 2019 to 2022 (*p* < 0.05). For DTaP-Hib, the respective reporting rates were 291.36 per 100,000 doses (20.00%), 586.80 per 100,000 doses (26.67%), 415.80 per 100,000 doses (17.78%), and 1200.30 per 100,000 doses (35.56%). For DTaP-IPV/Hib, the respective reporting rates were 97.81 per 100,000 doses (8.64%), 242.88 per 100,000 doses (21.36%), 243.07 per 100,000 doses (21.36%), and 778.13 per 100,000 doses (48.64%). The reporting rates of AEFIs following one dose and four doses of DTaP-IPV/Hib showed an increasing trend from 2019 to 2022 (*p* < 0.05).

There were statistically significant differences in the reporting rates of AEFIs among the three vaccines based on different numbers of vaccination doses (*χ^2^* = 125.96, 15.11, 133.06, *p* < 0.05). Both DTaP and DTaP-IPV/Hib had the highest reporting rates of AEFIs following four doses (*p* < 0.013), and the reporting rate of AEFIs following two doses and three doses of the two vaccines were higher than following one dose (*p* < 0.013). The reporting rates of AEFIs following four doses of DTaP-Hib was higher than that reported following one dose (*p* < 0.013). The reporting rate of AEFIs following two doses of DTaP-Hib was higher than DTaP (*χ^2^* = 5.15, *p* = 0.023). The reporting rates of AEFIs following one dose and three doses of DTaP-IPV/Hib were both lower than DTaP (*p* < 0.05) (Table 6).

**Table 6:** Reporting rates (per 10^5^ doses) of DTaP, DTaP-Hib, and DTaP-IPV/Hib AEFIs by vaccination dose in Linping District, 2019–2022.

Vaccination dose	*N* (Reporting rate)					*χ*^2^	*p*-value
	2019 Year	2020 Year	2021 Year	2022 Year	Total		
DTaP							
1	6 (67.97)	24 (340.28)	9 (152.72)	20 (456.31)	59 (225.57)	12.79	<0.001
2	6 (63.17)	29 (380.23)	23 (371.39)	25 (524.33)	83 (295.52)	24.00	<0.001
3	25 (239.88)	42 (527.17)	20 (310.80)	29 (571.88)	116 (393.51)	5.33	0.021
4	104 (776.24)	67 (760.41)	52 (623.50)	56 (901.77)	279 (759.00)	0.05	0.823
DTaP-Hib							
1	1 (112.49)	5 (472.14)	1 (170.07)	2 (361.66)	9 (291.36)	0.27	0.641
2	1 (186.92)	6 (1156.07)	2 (420.17)	3 (582.52)	12 (586.80)	0.11	0.800
3	0 (0.00)	3 (580.27)	3 (634.25)	2 (397.61)	8 (415.80)	0.72	0.432
4	1 (1538.46)	5 (1785.71)	8 (1388.89)	2 (485.44)	16 (1200.30)	3.39	0.279
DTaP-IPV/Hib							
1	1 (21.94)	3 (64.66)	7 (140.79)	8 (152.18)	19 (97.81)	5.36	0.023
2	9 (201.25)	11 (239.60)	13 (258.45)	14 (266.26)	47 (242.88)	0.44	0.505
3	3 (69.12)	13 (276.89)	21 (420.00)	10 (188.64)	47 (243.07)	1.94	0.164
4	6 (708.38)	41 (1168.76)	32 (678.54)	28 (598.29)	107 (778.13)	4.91	0.027

## 4 Discussion

The results of this study show that the combined reporting rates of AEFIs following DTaP, DTaP-Hib, and DTaP-IPV/Hib vaccinations in Linping District from 2019 to 2022 were 445.72, 536.29, and 306.13 per 100,000 doses, respectively. These rates were significantly higher than the average in the domestic regions such as Chengdu and Guangzhou (27, 28), as well as in foreign region such as Chile (17). The reporting rate of AEFIs from 2019 to 2022 following DTaP showed an increasing trend, reaching 631.36 per 100,000 doses in 2022. This could be attributed to increased public concern about vaccine safety after reports of issues with the DTaP vaccine efficacy from the Changchun Changsheng company in 2017, leading to heightened sensitivity in AEFI monitoring of vaccines containing DTaP components. The reporting rate of AEFIs following DTaP was higher than DTaP-IPV/Hib, with no statistically significant difference compared to DTaP-Hib, indicating that replacing DTaP with DTaP-Hib or DTaP-IPV/Hib will not be expected to increase the reporting of AEFIs.

In Linping District from 2019 to 2022, only one case of a serious AEFI was reported following DTaP vaccination, with no reports of serious AEFIs following DTaP-Hib and DTaP-IPV/Hib vaccinations. Regarding the types of AEFI reports, with 99.81% of DTaP and 97.78% of DTaP-Hib reports categorized as vaccine product-related reactions. The one reported coincidental event of DTaP and DTaP-Hib were diagnosed as epilepsy and bronchopneumonia, respectively. For DTaP-IPV/Hib, all reported AEFI cases were vaccine product-related reactions. The majority of reactions were mild. Among the vaccine product-related reactions for all three vaccines, the most common manifestations were local redness & swelling and local induration. The reporting rate of injection site redness & swelling for DTaP and DTaP-IPV/Hib were higher than the results of other studies (20, 29). The reporting rates of local redness & swelling and induration for DTaP and local induration for DTaP-IPV/Hib showed an increasing trend over the 4 years. Related studies have suggested that factors such as the dose of DTaP, shallow injection, incorrect administration, inadequate shaking before each dose, or previous freezing may increase the risk of local redness & swelling and induration (19). The reporting rates of local redness & swelling and induration for DTaP-IPV/Hib were lower than for DTaP, and the reporting rate of local induration for DTaP-Hib was lower than for DTaP, indicating that replacing DTaP with DTaP-Hib or DTaP-IPV/Hib will not be expected to increase the reporting of local reactions. There were only two reports of rash following DTaP vaccination, accounting for a small proportion of vaccine product-related reactions, with an reporting rate of 1.66 per 100,000 doses. This demonstrates that the preventive vaccination safety of vaccines containing DTaP components in Linping District is good. There were statistically significant differences in the reporting rates of local redness & swelling and induration between DTaP and DTaP-IPV/Hib, and both vaccines had the highest reporting rates for local redness & swelling and induration in the 2.6–5.0 cm category, consistent with previous research findings (28, 30). The reporting rate of local redness & swelling for DTaP-Hib in the 2.6–5.0 cm was also higher than that in the ≤2.5 and > 5.0 cm. The reporting rate of local redness & swelling for DTaP-IPV/Hib in the 2.6–5.0 cm category was lower than for DTaP, and the reporting rate of local induration for DTaP-Hib and DTaP-IPV/Hib in the 2.6–5.0 cm category were lower than for DTaP. The reporting rate of local redness & swelling for DTaP-Hib in the >5.0 cm was higher than that of DTaP, which may be related to the slow release of aluminum hydroxide adjuvant after vaccination, and the injection dose of DTaP-Hib was twice the capacity that of DTaP and DTaP-IPV/Hib, which could easily lead to more serious local reactions. It is suggested that vaccinators must vaccinate in strict accordance with the requirements to reduce the occurrence of local reactions.

The male-to-female ratio of reported AEFIs for DTaP, DTaP-Hib, and DTaP-IPV/Hib was 1.27:1, 1.05:1, and 0.95:1, respectively. The reporting rate of DTaP was higher than the levels reported in Chile (17), while the reporting rate of DTaP-IPV/Hib was lower than in other regions (28). The reporting rates of AEFIs for DTaP and DTaP-IPV/Hib were highest in the ≥18 months age group. Similarly, the proportion of AEFIs for DTaP-Hib in the ≥18 months age group was higher compared to the 6–17 months age group, also consistent with the ages at which vaccinations are scheduled. The reporting rates of AEFIs for DTaP was lowest in the first quarter, possibly due to the higher temperatures during the spring and summer seasons, resulting in a greater chance of skin exposure and increased detection of local redness & swelling or induration at the injection site. The DTaP-IPV/Hib had lower reporting rates of AEFIs in the 2nd, 3rd, and 4th quarters compared to DTaP, consistent with the overall reporting rates.

The reporting rates of AEFIs occurring within ≤1 day after vaccination were higher (>50%) than those occurring at 2–5, 6–15, and > 15 days, suggesting the need to continue improving on-site observation and acute reaction management in vaccination clinics to prevent the occurrence of severe cases. The reporting rates of AEFIs at 6–15 days following DTaP and DTaP-IPV/Hib vaccinations were higher than at >15 days, indicating the importance of educating parents about increased monitoring of their child’s health within the 2 weeks after vaccination, to promptly report and seek medical attention in case of abnormalities.

With the increase in the number of vaccination doses, specific reporting rates for both DTaP and DTaP-IPV/Hib gradually increased, with four doses having higher rates than 1–3 doses, consistent with previous research (28). The reporting rate of AEFIs following four doses of DTaP-Hib was also higher than that for one dose, indicating that the high reporting rate of AEFIs after booster vaccination may be related to the cellular immune response of the body, with the recipient repeatedly being stimulated at the injection site, leading to increased sensitivity to vaccine antigens or adjuvants (31). The reporting rates of AEFIs following one dose, two doses, and three doses of DTaP, and one dose of DTaP-IPV/Hib showed an increasing trend from 2019 to 2022, possibly because parents of younger infants may be more attentive to AEFIs and more likely to proactively report AEFI information. The reporting rate of AEFIs following two doses of DTaP-Hib was higher than DTaP, and the reporting rates of AEFIs following one dose and three doses of DTaP-IPV/Hib were both lower than DTaP, suggesting the need to strengthen monitoring of AEFIs after basic DTaP and DTaP-Hib vaccinations.

This study has certain limitations as it relies on passive surveillance for AEFI reporting, which may result in underreporting. Therefore, further efforts are needed to enhance training for vaccination clinic staff to improve the sensitivity and accuracy of AEFI monitoring.

## 5 Conclusion

The reported AEFIs to multi-component vaccines containing acellular pertussis components in Linping District were mainly mild reactions. DTaP-Hib and DTaP-IPV/Hib do not increase the reporting of AEFIs when replacing DTaP in vaccination. The safety of vaccines containing the DTaP component in Linping District is good, this may thereby reduce visits and improve immunization compliance and timeliness, and contribute to higher vaccination coverage. It is recommended to strengthen training for vaccination personnel, standardize vaccination work, and reduce the occurrence of local reactions. Additionally, consideration should be given to the dosing of DTaP-Hib, as the reporting rate of AEFIs has not decreased compared to DTaP. Therefore, further comparative studies evaluating the safety of multi-component vaccines and their component vaccines should continue to be a focus of future research, providing a basis for optimizing immunization strategies.

## Data availability statement

The original contributions presented in the study are included in the article/supplementary material, further inquiries can be directed to the corresponding authors.

## Ethics statement

Ethical approval was not required for the study involving humans in accordance with the local legislation and institutional requirements. Written informed consent to participate in this study was not required from the participants or the participants’ legal guardians/next of kin in accordance with the national legislation and the institutional requirements. Written informed consent was not obtained from the minor(s)’ legal guardian/next of kin, for the publication of any potentially identifiable images or data included in this article because it is a systematic review using preexisting, publicly published data.

## Author contributions

QC: Writing – original draft, Resources, Conceptualization. ChuZ: Methodology, Writing – original draft, Data curation. CY: Data curation, Formal analysis, Writing – review & editing. JZ: Supervision, Writing – review & editing, Formal analysis. JS: Data curation, Writing – review & editing. ChaZ: Data curation, Writing – review & editing. PY: Data curation, Writing – review & editing. TL: Formal analysis, Project administration, Software, Writing – review & editing. YX: Formal analysis, Methodology, Project administration, Writing – review & editing.

## References

[1] Nian X Liu H Cai M Duan K Yang X. Coping strategies for pertussis resurgence. Vaccines. (2023) 11:889. doi: 10.3390/vaccines11050889 37242993 PMC10220650

[2] Wu D Jing R Zheng H He K Li Y Yu W. Health and economic evaluation of vaccination against pertussis in China: a 40-year analysis. Value Health. (2023) 26:666–75. doi: 10.1016/j.jval.2022.10.011 36328326

[3] Sunarno AF Subangkit M Herna NS Kambang S Widoretno DF Tati F. Diphtheria serology in adults in Central Java and East Java, Indonesia: the importance of continuous diphtheria vaccination. Afr Health Sci. (2021) 21:1148–54. doi: 10.4314/ahs.v21i3.23 35222577 PMC8843289

[4] Boghani S Shah HD Fancy M Parmar T Bansal S Wanjari MB. A study on the characteristics and outcomes of reported diphtheria patients in a Western state in India. Cureus. (2023) 15:e35769. doi: 10.7759/cureus.35769 37025722 PMC10072171

[5] Xu Y Liu Y Du J Zheng W Liu S Zhang X. Seroepidemiology of tetanus in Hangzhou from 2009 to 2018. Hum Vaccin Immunother. (2020) 16:2670–6. doi: 10.1080/21645515.2020.1738170, PMID: 32460603 PMC7733938

[6] Callison C Nguyen H. Tetanus prophylaxis In: StatPearls. Treasure Island (FL): StatPearls Publishing. 2023. 32644434

[7] Dai P Wang Q Jia M Leng Z Xie S Feng L. Driving more WHO-recommended vaccines in the National Immunization Program: issues and challenges in China. Hum Vaccin Immunother. (2023) 19:2194190. doi: 10.1080/21645515.2023.2194190 37099400 PMC10158540

[8] Xu Y Xu E Liu S Zheng W Zhang X Du J. Seroepidemiology of pertussis in Hangzhou, China, during 2009-2017. Hum Vaccin Immunother. (2019) 15:2564–70. doi: 10.1080/21645515.2019.1608130, PMID: 31116637 PMC6930058

[9] Ma Y Sun Y Shen P Xu Y Zhao C Liu C. Genetic predisposition to adverse events in Chinese children aged 3-24 months after diphtheria, tetanus, acellular pertussis and *haemophilus influenzae* type b combined vaccination. Expert Rev Vaccines. (2022) 21:1923–8. doi: 10.1080/14760584.2022.2144239, PMID: 36328952

[10] Yin Z Zheng C Fang Q Wen T Wang S Li J. Comparing the pertussis antibody levels of healthy children immunized with four doses of DTap-IPV/Hib (Pentaxim) combination vaccine and DTaP vaccine in Quzhou, China. Front Immunol. (2023) 13:1055677. doi: 10.3389/fimmu.2022.1055677 36685526 PMC9852981

[11] Sharma H Marthak K Parekh S Pujari P Shewale S Desai S. A phase I study to evaluate safety and tolerability of DTaP-IPV + Hib vaccine in healthy adult volunteers in India. Vaccine X. (2023) 14:100300. doi: 10.1016/j.jvacx.2023.100300 37128477 PMC10148180

[12] Bauwens J de Lusignan S Weldesselassie YG Sherlock J Künzli N Bonhoeffer J. Safety of routine childhood vaccine coadministration versus separate vaccination. BMJ Glob Health. (2022) 7:e008215. doi: 10.1136/bmjgh-2021-008215 PMC9516064 36162867

[13] Liu B Cao B Wang C Sun T Miao Y Zhang S. Cost-minimization analysis of DTaP-IPV-Hib combination vaccine in China: a nationwide cross-sectional study. J Med Virol. (2023) 95:e28358. doi: 10.1002/jmv.28358 36448181

[14] Zhu F Zhuang C Chu K Zhang L Zhao H Huang S. Safety and immunogenicity of a live-attenuated influenza virus vector-based intranasal SARS-CoV-2 vaccine in adults: randomised, double-blind, placebo-controlled, phase 1 and 2 trials. Lancet Respir Med. (2022) 10:749–60. doi: 10.1016/S2213-2600(22)00131-X, PMID: 35644168 PMC9135375

[15] Di Pietrantonj C Rivetti A Marchione P Debalini MG Demicheli V. Vaccines for measles, mumps, rubella, and varicella in children. Cochrane Database Syst Rev. (2020) 4:CD004407. doi: 10.1002/14651858.CD004407 32309885 PMC7169657

[16] Gagliardi AM Andriolo BN Torloni MR Soares BG de Oliveira GJ Andriolo RB. Vaccines for preventing herpes zoster in older adults. Cochrane Database Syst Rev. (2016) 3:CD008858. doi: 10.1002/14651858.CD008858.pub4 26937872 PMC6516976

[17] Aguirre-Boza F San Martín PP Valenzuela BMT. How were DTP-related adverse events reduced after the introduction of an acellular pertussis vaccine in Chile? Hum Vaccin Immunother. (2021) 17:4225–34. doi: 10.1080/21645515.2021.1965424 34495813 PMC8828142

[18] HogenEsch H. Mechanisms of stimulation of the immune response by aluminum adjuvants[J]. Vaccine. (2002) 20:S34–9. doi: 10.1016/S0264-410X(02)00169-X 12184362

[19] Zimmermann P Curtis N. Factors that influence the immune response to vaccination. Clin Microbiol Rev. (2019) 32:e00084–18. doi: 10.1128/CMR.00084-18 30867162 PMC6431125

[20] Moro PL Perez-Vilar S Lewis P Bryant-Genevier M Kamiya H Cano M. Safety surveillance of diphtheria and tetanus toxoids and acellular pertussis (DTaP) vaccines. Pediatrics. (2018) 142:e20174171. doi: 10.1542/peds.2017-4171, PMID: 29866795 PMC6476554

[21] Ujiie M Tsuzuki S Suzuki M Ota M Suzuki T Nomoto H. Safety of diphtheria and tetanus toxoids and acellular pertussis (DTaP) vaccine in adults in Japan. Jpn J Infect Dis. (2021) 74:399–404. doi: 10.7883/yoken.JJID.2020.947 33518629

[22] Psihogios A Brianne Bota A Mithani SS Greyson D Zhu DT Fung SG. A scoping review of active, participant-centred, digital adverse events following immunization (AEFI) surveillance: a canadian immunization research network study. Vaccine. (2022) 40:4065–80. doi: 10.1016/j.vaccine.2022.04.103 35680501

[23] Sebastian J Gurumurthy P Ravi MD Ramesh M. Active surveillance of adverse events following immunization (AEFI): a prospective 3-year vaccine safety study. Ther Adv Vaccines Immunother. (2019) 7:251513551988900. doi: 10.1177/2515135519889000 PMC6873273 31799496

[24] Alguacil-Ramos AM Muelas-Tirado J Garrigues-Pelufo TM Portero-Alonso A Diez-Domingo J Pastor-Villalba E. Surveillance for adverse events following immunization (AEFI) for 7 years using a computerised vaccination system. Public Health. (2016) 135:66–74. doi: 10.1016/j.puhe.2015.11.010 26976484

[25] Pan X Lv H Liang H Wang Y Shen L Chen F. Surveillance on the adverse events following immunization with the pentavalent vaccine in Zhejiang, China. Hum Vaccin Immunother. (2022) 18:2021711. doi: 10.1080/21645515.2021.2021711, PMID: 35108152 PMC8986187

[26] World Health Organization (2018). Causality assessment of an adverse event following immunization (AEFI): User manual for the revised WHO classification (second edition). Licence: CC BY-NC-SA 3.0 IGO.

[27] Li L Yang RP Cai J Zheng JH. Evaluation of the safety of diphtheria, tetanus and acellular pertussis containing combination vaccines in Chengdu, 2015-2019. Zhonghua Yu Fang Yi Xue Za Zhi. (2020) 54:958–62. doi: 10.3760/cma.j.cn112150-20200417-00593 32907285

[28] Li Z Xu J Tan H Zhang C Chen J Ni L. Safety of pentavalent DTaP-IPV/Hib combination vaccine in post-marketing surveillance in Guangzhou, China, from 2011 to 2017. Int J Infect Dis. (2020) 99:149–55. doi: 10.1016/j.ijid.2020.07.019, PMID: 32795602

[29] Yang K Kim H Ortiz E Huoi C Kang J. Post-marketing safety surveillance of a childhood pentavalent diphtheria-tetanus-acellular pertussis-polio and *Haemophilus influenzae* type B (DTaP-IPV//Hib) vaccine in South Korea. Infect Dis Ther. (2023) 12:499–511. doi: 10.1007/s40121-022-00724-7, PMID: 36520326 PMC9925623

[30] Sun X Xu Y Tang F Xiao Y Wang Z Wang B. Immunogenicity and safety of concomitant administration of the chinese inactivated poliovirus vaccine with the diphtheria-tetanus-acellular pertussis (DTaP) vaccine in children: a multicenter, randomized, non-inferiority, controlled trial. Front Immunol. (2022) 13:905634. doi: 10.3389/fimmu.2022.905634 35958596 PMC9361845

[31] Li X Yang X Ning Z. Efficacy and safety of COVID-19 inactivated vaccine: a meta-analysis. Front Med. (2022) 9:1015184. doi: 10.3389/fmed.2022.1015184 PMC9676443 36419789

